# Biphasic activation of PI3K/Akt and MAPK/Erk1/2 signaling pathways in bovine herpesvirus type 1 infection of MDBK cells

**DOI:** 10.1186/1297-9716-42-57

**Published:** 2011-04-14

**Authors:** Liqian Zhu, Xiuyan Ding, Xiaofang Zhu, Songshu Meng, Jianye Wang, Hong Zhou, Qiangde Duan, Jie Tao, Dieter M Schifferli, Guoqiang Zhu

**Affiliations:** 1 College of Veterinary Medicine, Yangzhou University, Yangzhou 225009, China; 2 College of Life Science, Northwest A&F University, Yangling 712100, China; 3 Department of dermatology of Clinical medical school, Yangzhou University, Yangzhou 225009, China; 4 College of Bioscience and Biotechnology, Yangzhou University, Yangzhou 225009, China; 5 Department of Pathobiology, University of Pennsylvania School of Veterinary Medicine, Philadelphia, 19104, USA

## Abstract

Many viruses have been known to control key cellular signaling pathways to facilitate the virus infection. The possible involvement of signaling pathways in bovine herpesvirus type 1 (BoHV-1) infection is unknown. This study indicated that infection of MDBK cells with BoHV-1 induced an early-stage transient and a late-stage sustained activation of both phosphatidylinositol 3-kinase (PI3K)/Akt and mitogen activated protein kinases/extracellular signal-regulated kinase 1/2 (MAPK/Erk1/2) signaling pathways. Analysis with the stimulation of UV-irradiated virus indicated that the virus binding and/or entry process was enough to trigger the early phase activations, while the late phase activations were viral protein expression dependent. Biphasic activation of both pathways was suppressed by the selective inhibitor, Ly294002 for PI3K and U0126 for MAPK kinase (MEK1/2), respectively. Furthermore, treatment of MDBK cells with Ly294002 caused a 1.5-log reduction in virus titer, while U0126 had little effect on the virus production. In addition, the inhibition effect of Ly294002 mainly occurred at the post-entry stage of the virus replication cycle. This revealed for the first time that BoHV-1 actively induced both PI3K/Akt and MAPK/Erk1/2 signaling pathways, and the activation of PI3K was important for fully efficient replication, especially for the post-entry stage.

## Introduction

Bovine herpesvirus-1 (BoHV-1) classified into the subfamily Alphaherpesvirinae of Herpesviridae [1], is a pathogen of major economic importance in the cattle industry worldwide. The virus is a causative agent of respiratory tract (infectious bovine rhinotracheitis) and genital tract (infectious pustular vulvovaginitis and balanoposthitis) infections, as well as conjunctivitis, central nervous disorders, and abortions in pregnant cows [2-4]. Inflammation and necrosis of respiratory epithelia and immunosuppression caused by the virus infection often lead to increased susceptibility to secondary viral and bacterial infections, which results in enhanced morbidity and mortality [5]. Thus, the worldwide distributed virus causes serious economic loss for the cattle industry.

Phosphatidylinositol 3-kinases (PI3K) are an important family of cellular, heterodimeric lipid kinase (Class I, II and III) that consist of a regulatory subunit (p85) and a catalytic subunit (p110) [6,7]. After activation, the p110 subunit of PI3K phosphorylates the lipid substrate phosphatidylinositol 4, 5-bisphosphate (PIP2) to produce phosphatidylinositol 3, 4, 5-trisphosphate (PIP3) [8]. This molecule serves as a potent second messenger to regulate phosphorylation of a wide variety of signal transduction proteins, including Akt which is activated by phosphorylation at Thr308 and Ser473 [9]. A number of molecules are directly or indirectly regulated by Akt, to carry out PI3K-regulated responses such as cell survival, growth, proliferation, angiogenesis, metabolism, and migration [10].

The members of the mitogen-activated protein kinase (MAPK) superfamily respond to diverse cellular stimuli through transducing signals from the cell membrane to the nucleus [11]. Extracellular signal-regulated kinases 1 and 2 (Erk1/2) are members of MAPK family, which regulate a wide range of cellular functions including cell proliferation, transformation, differentiation, cell survival and death [12].

An increasing number of viruses have been found to gain control of key cellular signaling pathways including PI3K/Akt and MAPK/Erk1/2. For example, the PI3K/Akt pathway is found to be required for the efficient replication of influenza A virus, human cytomegalovirus and Junín virus [6,13,14]. Furthermore, activation of MAPK/Erk1/2 pathway occurred during the virus infection for Coxsackievirus B3, Human immunodeficiency virus type 1, hepatitis B virus, Kaposi's sarcoma-associated herpesvirus and human cytomegalovirus [15-19]. Inhibition of corresponding pathways leads to the reduction of virus yield, indicating that trigger of certain signaling pathway(s) is necessary for virus multiplication. However, little is known regarding the signal pathways involved in BoHV-1 infection of MDBK cells.

In this study, whether BoHV-1 infection of MDBK cells involved the activation of PI3K/Akt and/or MAPK/Erk1/2 pathways was investigated. We observed that BoHV-1 infection of MDBK cells led to a biphasic activation of PI3K/Akt and MAPK/Erk1/2 pathways and the activation of PI3K was required for fully efficient replication, especially for the post-entry step.

## Materials and methods

### Viruses and cell cultures

The Colorado1 strain of BoHV-1 and MDBK cells were supplied by Dr Leonard J Bello, University of Pennsylvania, USA [20]. MDBK cells were maintained in Dulbecco's modified Eagle's medium (DMEM) (Gibco BRL) supplemented with 10% horse serum, in an incubator at 37°C containing 5% CO2. Virus was propagated in large amount in MDBK cells. The virus stocks were titrated and stored at -80°C until use.

### Antibodies and reagents

Rabbit polyclonal antibodies recognizing phospho-Akt (Ser473) and Akt, rabbit monoclonal antibody recognizing phospho-p44/42 MAPK (Erk1/2) (Thr202/Tyr204) were purchased from Cell Signaling Technology Technology, **Beverly, Mass**. Mouse monoclonal β-actin antibody was obtained from Sigma, **St. Louis, MO**. Horseradish peroxidase (HRP)-conjugated anti-rabbit IgG and anti-mouse IgG were purchased from Santa Cruz Biotechnology, Inc., **Santa Cruz, Calif**. The PI3K-specific inhibitor LY294002 and MEK1/2 inhibitor U0126 were supplied by Cell Signaling Technology.

### Determination of the virus titer

MDBK cells seeded in 96-well microplates were grown to approximately 50% confluence. Serial 10-fold dilutions of virus stocks were prepared in DMEM and 100 μL of each dilution was added to the wells. After 72 h incubation, when the cytopathic effect (CPE) of BoHV-1 infection was evident, the total number of CPE positive wells was counted by using an inverted microscope and the result is expressed as TCID_50 _calculated using Reed-Muench formula [21].

### Virus infection

When the cells in 6 mm dishes were grown to 70%-80% confluence, they were subjected to serum starvation for 24 h. The growth arrested cells were infected at a multiplicity of infection (MOI) of 2 with BoHV-1 or were mock infected with the same medium but without virus. 1 h post-infection (pi), cells were washed with phosphate-buffered saline (PBS) and then cultured in fresh medium for various time lengths as indicated.

For inhibitor experiments, cells were pretreated with the PI3K inhibitor ly294002, or the MEK inhibitor U0126, for 1 h. Cells were then infected with the virus for 1 h, washed with PBS, and placed in serum-free media containing fresh inhibitor unless otherwise specified. The 1000 × stock of inhibitors were prepared in DMSO (Shanghai Sangon Biological Engineering Technology & Services Co., Ltd). DMEM containing DMSO was used for the mock treatment unless otherwise specified.

### Solubilization of cells, and Western blotting analysis

After the BoHV-1 infection or mock infection for the length of time indicated, mono-layers cells were washed with PBS and lysed with 300 μL lysis buffer as described elsewhere [22]. Lysates were cleared by centrifugation for 15 min at 12 000 *g*. Samples were resolved by SDS-PAGE under reducing conditions, and transferred onto nitrocellulose membranes (Bio-Rad). Membranes were blocked for 1 h at room temperature with nonfat dry milk solution (5% in Tris-buffered saline) containing 0.1% Tween 20. Blots were incubated overnight at 4°C with primary antibodies followed by incubation for 1 h with the secondary antibody (horseradish peroxidase congugated). After extensive wash, the immunoreactive bands were detected by enhanced chemiluminescence (ECL) (Pierce). Subsequently, the same membrane was stripped and reprobed for total protein loading using an anti-Akt or anti-β-actin antibody.

### UV-irradiation of BoHV-1

To inactivate BoHV-1with UV-irradiation, the virus stocks were dispersed in a 10-cm tissue culture dishes, and directly placed under a UV lamp (20W) for 30 min. Complete inactivation of the virus was confirmed by the fact that CPE was not produced in MDBK cells infected with the treated virus.

### Analysis the effect of inhibitors Ly294002 and U0126 on the virus replication

To analyze whether inhibition of PI3K/Akt and MAPK/Erk1/2 pathway by corresponding inhibitor ly294002 and U0126 affected the virus replication, MDBK cells seeded in 24-well plates were pre-incubated with the inhibitors or mock pretreated for 1 h at 37°C, and then infected with 50TCID_50 _BoHV-1 for 1 h. Following extensive wash with PBS, the cells were placed in DMEM with or without fresh inhibitors. After 24 h incubation the cells were subjected to freezing-thawing and the virus yield was titrated in 96-well microplates with MDBK cells and the result was expressed as TCID_50_.

### Effect of PI3K inhibitor on the virus infection at the virus binding, entry and post-entry stage

To evaluate the effect of PI3K specific inhibitor Ly294002 on BoHV-1 attachment, MDBK cells seeded in 24-well plates were incubated in the absence or presence of 10 μM Ly294002 for 1 h at 37°C. The virus in ice-cold DMEM containing fresh inhibitor was then allowed to adsorb to the target cells for 1 h at 4°C. After extensive washing and immediately freezing-thawing, the cell-associated virus was titrated. For the entry assay, the procedure was similar to that of the binding assay, except that the entry process occurred at 37°C, and further incubation of about 24 h was needed. For the post-entry assay, after infection with 50TCID_50 _virus and subsequent washing, medium in the absence or presence of 10 μM Ly294002 was placed and incubated for 24 h at 37°C. After two rounds of freezing-thawing, the progeny virus was titrated.

## Result

### BoHV-1 infection of MDBK cells leads to the activation of PI3K/Akt signal pathway

**Figure 1:**
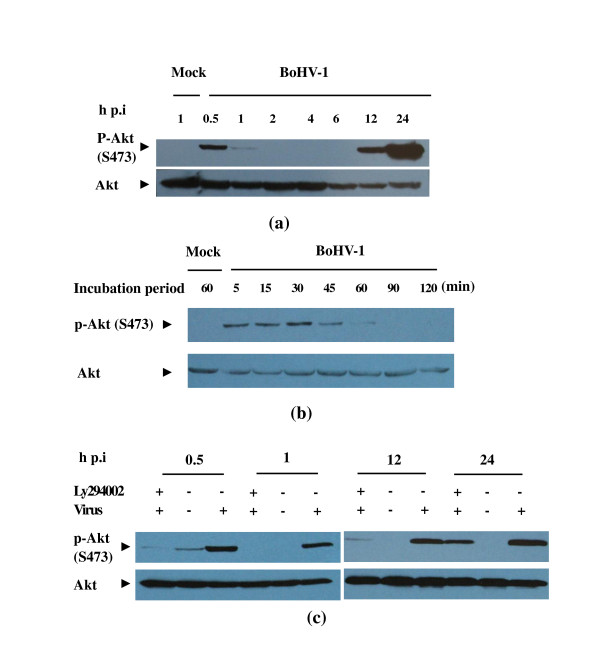
**BoHV-1 infection of MDBK cells induced Akt phosphorylation via a PI3K-dependent pathway**. (a) Time course for BoHV-1 stimulation and Akt phosphorylation. Growth arrested cells were infected with BoHV-1 and processed for Western blotting at the pi time indicated. Mock infected cells were used as a control. (b) Time course for BoHV-1 stimulation and Akt phosphorylation at the early time. (c) PI3K specific inhibitor Ly294002 suppressed Akt phosphorylation induced by the virus infection. Cells preincubated with medium in the absence of Ly294002 and then mock infected or infected with the virus were used as the negative and positive control, respectively. Growth arrested MDBK cells were pretreated with Ly294002 and infected with BoHV-1 at an MOI of 2 in the presence of the inhibitor, and then processed for Western blotting at the pi time indicated with phosphor-Akt antibody. Western blotting was performed with Akt antibody to detect the protein loading. Each experiment was repeated three times, and representative results are shown.

To exploit the cellular pathways involved in BoHV-1 infection of MDBK cells, we first examined whether virus infection leads to the activation of PI3K/Akt pathway by testing the level of phosphorylated Akt as determined by the others [6,23]. Serum starved MDBK cells were mock-infected or infected with BoHV-1 at a MOI of 2. Cell lysates prepared at the time indicated were subjected to Western blotting using the antibody specific for phospho-Akt (Ser473). The phosphorylated on Ser 473 has been shown to correlate with Akt kinase activity thus provides a convenient, reliable method to analyze Akt activity [13,24]. Western blotting analysis indicated that Akt was activated in a two-tiered manner following BoHV-1 infection (Figure 1a, top panel). The first tier of Akt activation occurred at the early time (0.5 and 1 h pi) of the virus infection (Figure 1a). From 2 h pi Akt activation disappeared, then after a certain intervals the second tier appeared and the elevated phosphorylation was sustained for the remainder of the infection (Figure 1a, 12 and 24 h pi). The changes in Akt phosphorylation were not due to the variation of total protein loading (Figure 1a bottom panel). Subsequently, the kinetics of Akt phosphorylation at the early time of the virus infection was extensively examined. As indicated in Figure 1b, exposure of MDBK cells to BoHV-1 could active Akt as early as 5 min, and it peaked at 0.5 h pi and remained for 1 h pi at a high level. Therefore 0.5 h and 1 h were chose as the early infection time points, 12 h and 24 h were chose as the late infection time points for the following experiment unless otherwise specified.

In view of the fact that Akt could be activated by either PI3K-dependent or -independent ways [6,25], we next analyzed the role of PI3K in Akt phosphorylation following BoHV-1 infection. To do this, Ly294002, a potent and specific inhibitor of PI3K was used [26]. MDBK cells infected with the virus in the presence of 10 μM Ly294002 were processed for immunoblot assay. As can be seen in Figure 1c, the drug inhibited BoHV-1-mediated Akt phosporylation at both early and late stages of the virus infection without affecting the level of total Akt expression. Though the activation could not be completely blocked at 24 h pi, the amount of the phosphorylated Akt significantly declined compared to the control. These results indicate that the virus inducing Akt activation was mediated by PI3K. We concluded that BoHV-1 infection induces the activation of PI3K/Akt signal pathway at both early and late step.

### BoHV-1 infection leads to the activation of MAPK/Erk1/2 signal pathway

**Figure 2:**
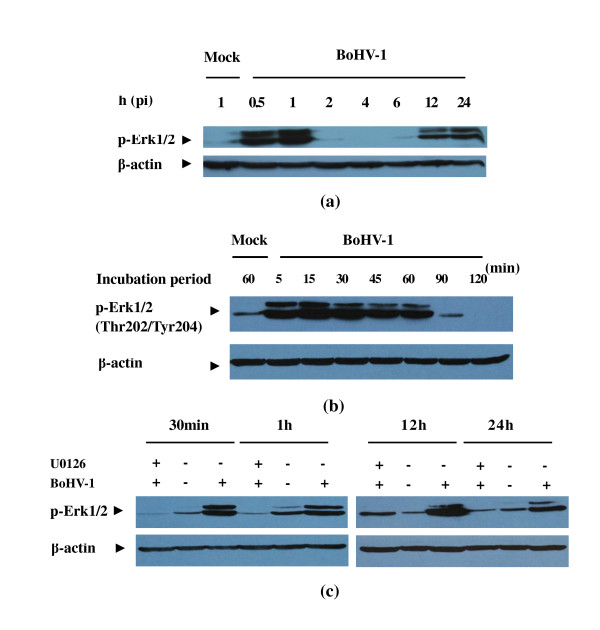
**BoHV-1 infection of MDBK cells induced the activation of MAPK/Erk1/2 pathway**. (a) Time course for BoHV-1 stimulation and Erk1/2 phosphorylation. Growth arrested cells were infected with BoHV-1 and processed for Western blotting at pi time indicated. Mock infected cells were used as a control. (b) Time course for BoHV-1 stimulation and Erk1/2 phosphorylation at the early time. (c) MEK specific inhibitor U0126 inhibited the virus induced Erk1/2 phosphorylation. Cells preincubated with medium in the absence of U0126 and then mock infected or infected with virus were used as the negative and positive control, respectively. Western blotting was performed with β-actin antibody to detect the protein loading. Growth arrested MDBK cells were pretreated with Ly294002 and infected with BoHV-1 at an MOI of 2 in the presence of the inhibitor, and then processed for western blotting at the time indicated with phospho-p44/42 MAPK (Erk1/2) (Thr202/Tyr204) antibody. Each experiment was repeated three times, and representative results are shown.

MAPK (Erk1/2) signaling pathway plays pivotal roles in regulating many cellular programs such as cell proliferation, differentiation, motility, and death. Therefore, we investigated whether BoHV-1 could induce intracellular Erk1/2 phosphorylation during the course of infection. Growth arrested MDBK cells were either infected with BoHV-1 at a MOI of 2 or mock infected for the time intervals indicated. Cell lysates were processed for Western blotting. Immunoblotting was conducted using antibody specific for phosphorylated Erk1/2 (Thr202/Tyr204). The data indicated that Erk1/2 was activated in a two-tiered manner following BoHV-1 infection (Figure 2a top panel). Indeed, BoHV-1- induced Erk1/2 phosphorylation occurred at both the early and late stages of the virus infection. The activation appeared at the early stage, declined to the basal level at 2 h pi then after several hours it was reactivated and remained high to the end of the experiment (Figure 2a, 12 and 24 h pi). Here, β-actin was used as a control for the total protein loading. Obviously, the kinetics of Erk1/2 phosphorylation was not due to the variation of total protein loading (Figure 2a, bottom panel). Subsequently, the kinetics of BoHV-1- activated Erk1/2 at early stage of the virus infection was extensively determined. As indicated in Figure 2b, exposure of MDBK cells to BoHV-1 activated Erk1/2 as early as 5 min and remained at a high level until 1 h pi. At 1.5 h pi, the phosphorylated Erk1/2 decreased to the basal level. Therefore 0.5 h and 1 h were chose as the early infection time points, 12 h and 24 h were chose as the late infection time points for the following investigation unless otherwise specified.

MEK1/2 MAPK or Erk kinases, are dual-specificity protein kinases which function in a mitogen activated protein kinase cascade controlling cell growth and differentiation. Thus, whether MEK1/2 acted as an upstream activator for BoHV-1-induced activation of Erk1/2 was investigated by using U0126, a highly potent and specific inhibitor of MEK 1/2. The results indicated that treatment of MDBK cells with a MAPK-specific inhibitor U0126 leaded to the significant suppression of BoHV-1-mediated Erk1/2 phosphorylation (Figure 2c). These results confirmed that BoHV-1 infection of MDBK cells induced the activation of MAPK/Erk1/2 signaling pathway.

### UV-irradiated BoHV-1 activated both PI3K/Akt and MAPK/Erk1/2 signaling pathways at the early stage

**Figure 3:**
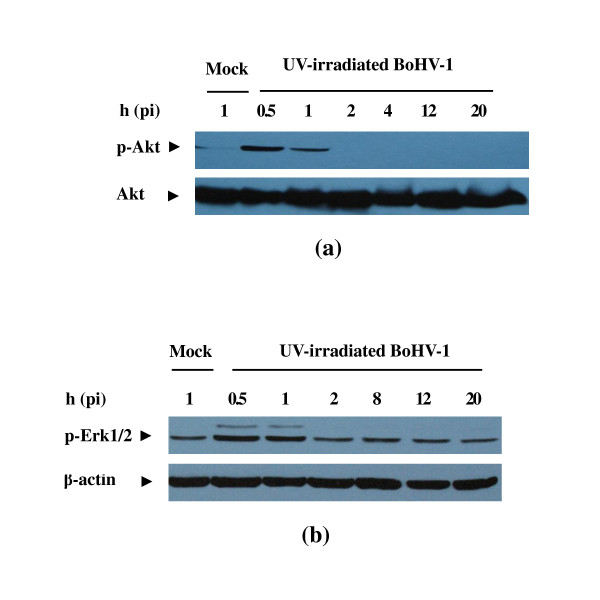
**UV-irradiation inactivated virus induced activation of Akt (a) and Erk1/2 (b) at the early time**. Serum-starved MDBK cells were mock infected or infected with UV-irradiated BoHV-1 and then processed for Western blotting at the time point indicated with phosphor-Akt antibody and phospho-p44/42 MAPK (Erk1/2) (Thr202/Tyr204) antibody. Western blotting was performed with Akt antibody (a) and β-actin antibody (b) to detect the protein loading. Each experiment was repeated three times, and representative results are shown.

To further elucidate the mechanisms of BoHV-1 mediated Akt and Erk1/2 activation, MDBK cells were infected by UV-irradiated BoHV-1 virus. Such inactivated virus fails to express viral proteins due to the formation of thymidine dimers, which prevent the transcription of viral genes, but this does not interfere with the virus capacity for receptor binding and endocytosis into host cells [27]. Here, inactivation of the virus was ascertained as determined by plaque assay (data not shown). As showed in Figure 3, the UV-irradiated virus also led to the first tier phosphorylation of Akt and Erk1/2, but the second tier event completely disappeared, suggesting that virus-cell interaction would be responsible for the early step activation. Together with the data showed in Figure 1a and Figure 2a, we inferred that the late phasic activation of the two signaling pathways was associated with viral genes expression or protein synthesis.

### PI3k/Akt pathway played significant role in the virus production

**Figure 4:**
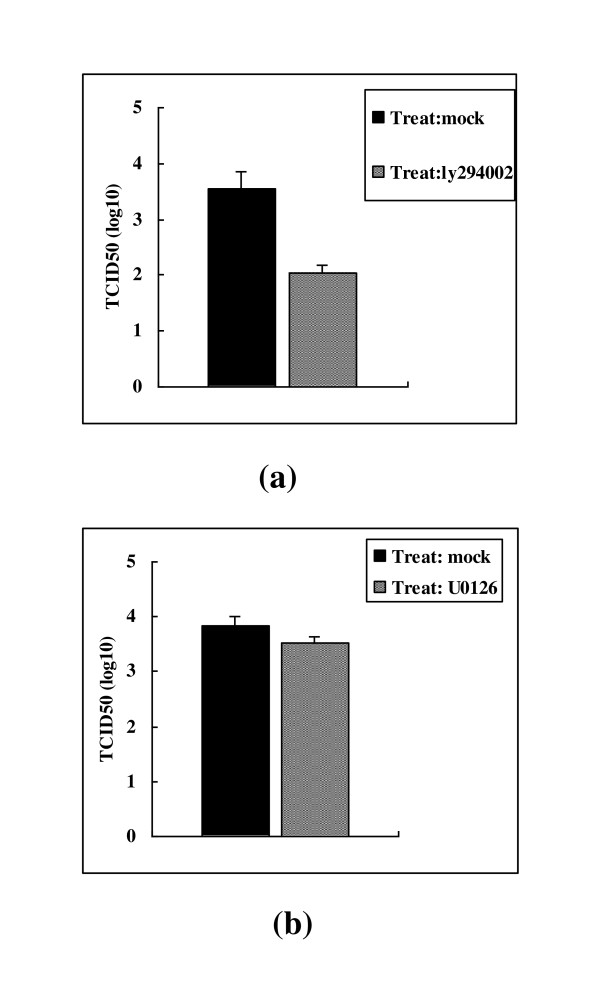
**Virus production was significantly reduced by the inhibition of PI3k/Akt pathway (a) but not by MAPK/Erk1/2 pathway (b)**. (a) Growth arrested MDBK cells mock pretreated or pretreated with 10 μM Ly294002 were infected with BoHV-1 virus in the medium with or without Ly294002. After 24 h incubation the virus production were titrated. (b) Growth arrested MDBK cells mock pretreated or pretreated with 10 μM U0126 were infected with BoHV-1 virus in the medium with or without U0126. After 24 h incubation the virus production were titrated. Experiments were repeated three times, and the error bars indicate the standard deviations of three independent experiments.

The above results indicate that BoHV-1 virus infection resulted in PI3K/Akt and MAPK/Erk1/2 signaling pathway activation. Therefore, the role of the activated signaling in regulating the virus multiplication was further investigated. To determine whether the activation of PI3K/Akt by BoHV-1 played a role in virus replication, we examined the effect of PI3K specific inhibitor Ly294002 on virus production in MDBK cells. The data indicated that with the treatment of 10 μM Ly294002 the virus yield reduced a titer of 1.5 log in comparison with the control samples (Figure 4a). To determine whether the activation of MAPK/Erk1/2 by BoHV-1 played a role in virus replication, U0126, a selective inhibitor of MAPK/Erk1/2 pathway, which inhibits MEK, immediate upstream activator of Erk 1/2 was employed. However, treatment of the cells with 10 μM U0126 resulted in minor effect on the virus production (Figure 4b). In addition, the concentration of U0126 at 10 μM could completely block MAPK/Erk1/2 activation (Figure 2c). Taken together, these data indicated that PI3K/Akt pathway was important for the virus production. In addition, the inhibition of MAPK/Erk1/2 pathway was not enough to reduce the virus yield, though it was also activated during virus infection.

### PI3k/Akt pathway was important for the virus replication at the post-entry stage

**Figure 5:**
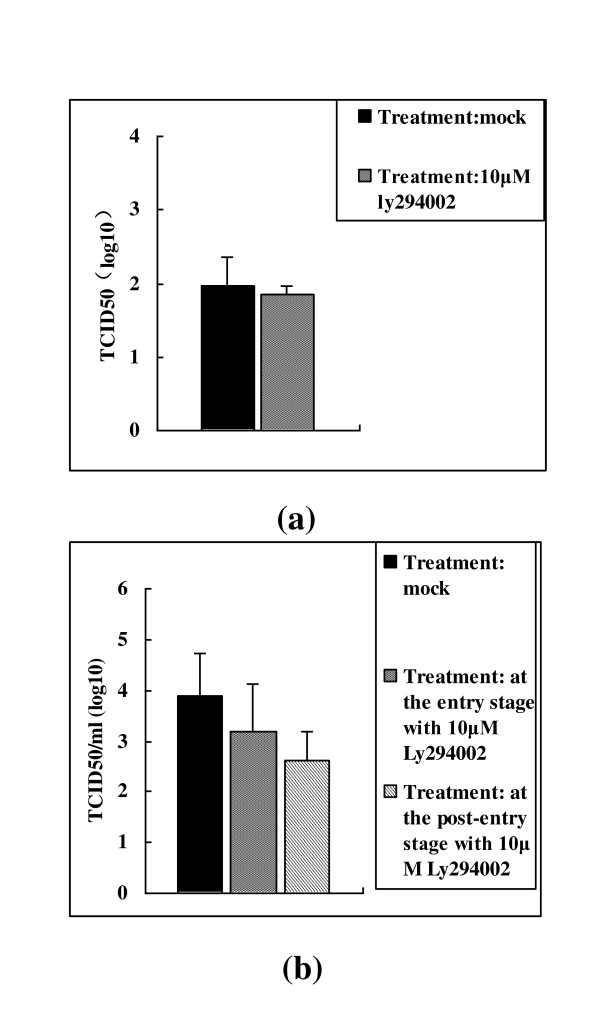
**Effect of PI3K inhibition with Ly294002 on the virus infection of MDBK cells at the virus binding stage (a), entry and post-entry stage (b)**. (a) Binding of the virus to the MDBK cells pretreated with the inhibitor or mock pretreated were determined with virus titration. (b) BoHV-1 produced in MDBK cells treated with Ly294002 at the viral entry and post-entry stages were titrated. Experiments were repeated three times, and the error bars indicate the standard deviations of three independent experiments.

In view of the importance of the PI3K/Akt pathway for BoHV-1 replication, the potential for the production of the virus at virus binding, entry and post-entry stage was evaluated, respectively. The results indicate that inhibition of PI3K at the binding stage and entry stage had no or minor effect on virus replication, while when it was inhibited at the post-entry stage the virus yield reduced significantly with a 1.3-log reduction in viral titer (Figure 5). These data suggested that activation of PI3K was required for the fully efficient replication at the post-entry stage.

## Discussion

Over the course of evolution, viruses have developed various strategies that modulate a variety of host cell signaling pathways to establish a moderate environment favourable for their survival or infection. In particular, delaying or evading cell apoptosis affords great advantages to the virus, facilitating virus replication, the spreading of progeny virus to neighboring cells and providing the protection for the progeny virus against cellular enzymes [28]. PI3K/Akt and MAPK/Erk1/2 are such pathways that have attracted much interest due to its central role in the regulation of cell death, proliferation and survival.

In the present data we showed that the virus infection induced biphasic activation of PI3K/Akt and MAPK/Erk1/2 pathways in MDBK cells. Once exposed to BoHV-1, Akt and Erk1/2 were transiently phosphorylated, disappearing within a short period; after several hours another tier of persistent activation was observed (Figure 1a and Figure 2a). To preclude the possibility that cellular components in the virus stocks could activate the signaling pathways, the virus was pelleted by ultracentrifugation at 20,000 rpm (Beckman SW28 rotor) for 1 h as described elsewhere [29]. The pelleted virus could enhance the level of both phosphoralated Akt and Erk1/2, however, the supernatant could not (data not shown). Take together the data demonstrate that BoHV-1 could active Akt and Erk1/2 in the infection of MDBK cells. In addition, we showed that inhibition of PI3K resulted in a significant reduction of the progeny virus production.

Further investigation revealed that sustaining activation of PI3K at the post-entry stage was important for virus replication. Though the virus titer reduced 0.6 log compared to the control when it was inhibited at the virus entry stage (Figure 5b), we inferred that the inhibition of PI3K had minor effect on the virus entry. Since the PI3K inhibitor Ly294002 is a cell permeable compound, when it was used in the entry stage, the intracellular residues had some effect on the subsequent stage of the virus replication. Studies currently in progress would show if this was indeed the case. Thus these data suggest that PI3K activation played an important role in fully efficient replication, especially at the post-entry stage. This study provides new insight into our understanding of the interplay between virus BoHV-1 and MDBK cells signaling pathways induced upon infection.

Recent work indicated that many virus exploit host cell signaling pathways to facilitate various steps of virus infection. The PI3K/Akt pathway was proved to be critical in regulating vesicular trafficking for Ebola virus at the entry step. In addition, blocking phosphorylation of Akt at the early step nearly aborted virus replication [30]. Influenza A virus triggered PI3K/Akt pathway activation only at the late phase in infection of human lung carcinoma cells (A549), and the activation has been shown to be required for efficient virus replication [6]. This report indicates that BoHV-1 infection of MDBK cells led to biphasic activation of PI3K/Akt and MAPK/Erk1/2 pathways and Inhibition of PI3K/Akt pathway greatly reduced the virus production (Figure 4a). This evidence was the first to imply that activation of PI3K/Akt is important for BoHV-1 to complete its life cycle. For many viruses, the MAPK/Erk1/2 pathway had been proved to take part in the regulation of gene expression and replication. For example, a bi-phasic Erk1/2 is activated to facilitate Coxsackievirus B3 and human cytomegalovirus infection [19,15]. Treatment of the cells with MAPK stimulators, such as serum and phorbol myristate acetate to activate MAPK/Erk1/2 signaling pathway could enhanced the replication and infectivity of HIV-1 and BK polyomavirus virus [16,31]. However, inhibition of MAPK/Erk1/2 pathway had a minor effect on BoHV-1 replication, though biphasic activation was also induced by the virus (Figure 4b and Figure 2a). To explain this, we speculated that during the process of the virus infection once the signaling pathway was inhibited simultaneously or subsequently, another signaling pathway(s) was (were) activated which could replenish the function of MAPK/Erk1/2 pathway. However, further research on this matter is needed.

Shortly after exposure of cells to UV-irradiated virus, which was capable of receptor binding and endocytosis, the early PI3K/Akt and MAPK/Erk1/2 pathways activation was induced, but it was insufficient to induce the late stage phosphorylation (Figure 3). The mechanism for the rapid activation of the two pathways by BoHV-1 was unknown. These activations may be induced by direct virus and cellular receptor(s) and/or co-receptor(s) interaction, such as that seen in the HIV-1 activation of Erk [32], or by exposure to a viral protein such as the HIV-1 Tat protein [33]. Furthermore, phosphorylation of Akt and Erk1/2 with high level was observed as early as 5 min postinfection strongly indicating that the virus could actively induce the signaling pathways and virus replication was not required for these inductions. Inversely, UV-irradiated virus, incapable of replication, could not active the late-phase activation of the two pathways. Obviously the viral gene expression was responsible for this event as reported in influenza A virus and human cytomegalovirus infection [6,13].

In summary, our investigation indicates that PI3K/Akt and MAPK/Erk1/2 signaling pathways are induced as a consequence of BoHV-1 infection of MDBK cells. In particular, the activation of PI3K was required for fully efficient replication, especially at the late step. It enhanced our knowledge on the mechanism of the virus infection. The complex interaction between the virus and cellular components during the course of BoHV-1 infection needs to be more clearly defined in the future by focusing on the signaling pathway.

## Competing interests

The authors declare that they have no competing interests.

## Authors' contributions

LQZ carried out all of the experiments, participated in the data collection and analysis, and the prepared the manuscript. XYD participated in the design of the study and draft of the manuscript. XFZ participated in cell culture, data collection and analysis. SSM participated in the design of the study and data analysis. JYW participated in data collection. HZ participated in cell culture. QDD participated in cell culture. JT participated in cell culture. DMS participated in manuscript revision. GQZ conceived of the study, and participated in its design and coordination. All authors read and approved the final manuscript
